# Protective efficacy of a broadly cross-reactive swine influenza DNA vaccine encoding M2e, cytotoxic T lymphocyte epitope and consensus H3 hemagglutinin

**DOI:** 10.1186/1743-422X-9-127

**Published:** 2012-06-27

**Authors:** Bin Wang, Hai Yu, Fu-Ru Yang, Meng Huang, Ji-Hong Ma, Guang-Zhi Tong

**Affiliations:** 1 Division of Swine Infectious Diseases, Shanghai Veterinary Research Institute, Chinese Academy of Agricultural Sciences, Shanghai, 200241, China; 2 State Key Laboratory of Veterinary Biotechnology, Harbin Veterinary Research Institute, Chinese Academy of Agricultural Sciences, Harbin, Heilongjiang, 150001, China; 3 College of Veterinary Medicine, Northeast Agricultural University, Harbin, Heilongjiang, 150030, China

**Keywords:** Swine influenza, DNA vaccine, Consensus HA, M2e, CTL epitope

## Abstract

**Background:**

Pigs have been implicated as mixing reservoir for the generation of new pandemic influenza strains, control of swine influenza has both veterinary and public health significance. Unlike human influenza vaccines, strains used for commercially available swine influenza vaccines are not regularly replaced, making the vaccines provide limited protection against antigenically diverse viruses. It is therefore necessary to develop broadly protective swine influenza vaccines that are efficacious to both homologous and heterologous virus infections. In this study, two forms of DNA vaccines were constructed, one was made by fusing M2e to consensus H3HA (MHa), which represents the majority of the HA sequences of H3N2 swine influenza viruses. Another was made by fusing M2e and a conserved CTL epitope (NP147-155) to consensus H3HA (MNHa). Their protective efficacies against homologous and heterologous challenges were tested.

**Results:**

BALB/c mice were immunized twice by particle-mediated epidermal delivery (gene gun) with the two DNA vaccines. It was shown that the two vaccines elicited substantial antibody responses, and MNHa induced more significant T cell-mediated immune response than MHa did. Then two H3N2 strains representative of different evolutional and antigenic clusters were used to challenge the vaccine-immunized mice (homosubtypic challenge). Results indicated that both of the DNA vaccines prevented homosubtypic virus infections completely. The vaccines’ heterologous protective efficacies were further tested by challenging with a H1N1 swine influenza virus and a reassortant 2009 pandemic strain. It was found that MNHa reduced the lung viral titers significantly in both challenge groups, histopathological observation showed obvious reduction of lung pathogenesis as compared to MHa and control groups.

**Conclusions:**

The combined utility of the consensus HA and the conserved M2e and CTL epitope can confer complete and partial protection against homologous and heterologous challenges, respectively, in mouse model. This may provide a basis for the development of universal swine influenza vaccines.

## Background

Swine influenza virus (SIV), member of genus Influenza A virus of the family Orthomyxoviridae, is a common and important causative pathogen involved in the porcine respiratory disease. Mortality of SIV-infected pigs is low, but morbidity may approach 100%. Clinical signs of swine influenza include high fever, lethargy, anorexia, coughing, labored breathing and nasal discharge. Synergistic or secondary infections with opportunistic organisms may increase the severity of clinical disease [1]. Beyond the veterinary implications, influenza virus infections in pigs also present an important public health risk. Since pigs express sialic acid receptors for both mammalian and avian strains of influenza viruses on their tracheal epithelial cells [2], they could potentially serve as “mixing vessels” for the generation of new reassortant strains of influenza viruses that have pandemic capacity. The recently emerged 2009 pandemic H1N1 which resulted in over 18,449 deaths is an example (http://www.who.int/csr/don/2010_08_06/en/index.html). Genetic analyses revealed that this pandemic H1N1 influenza virus is a triple reassortment of multiple strains of viruses circulating in the North America and Eurasia swine population [3,4]. Therefore, SIV-infection control would be of benefit to both reduce the economic losses of swine industry and human health.

Vaccination is considered to be the most effective method to control SIVs. Currently, commercially available swine influenza vaccines are adjuvanted, whole-virus killed vaccines. Although the vaccines reduce the severity of disease and the extent of virus shedding in pigs after challenge, they do not provide consistent protection from infection [5]. The vaccines function by targeting the surface glycoprotein hemagglutinin (HA), the most variable influenza virus antigen. Protective efficacy depends on the antigenic match degree between vaccine and circulating strains. However, influenza viruses continuously evolve by increasing the mutations in epitopes (antigenic drift) or by reconstituting the genome with other strains (antigenic shift). The increased incidence of avian-like or human-like SIV reassortants, which exhibit great genetic diversity and thus antigenic diversity with classical SIVs, has been documented [6-8], resulting in the “antigenic mismatch” between vaccine and the circulating strains. In addition, each of the dominant subtypes circulating in swine population worldwide, i.e. H1N1, H3N2 and H1N2, has developed multiple genetic clusters based on the phylogenetic analysis of HA genes [9-12]. Previous studies have demonstrated that some viruses in different clusters showed only limited cross-reactivity [10,12], suggesting that the genetic and antigenic heterogeneities within each subtype may reduce the vaccines’ effectiveness [13-16]. Vaccines that induce broad protective immunity against diverse SIV clusters and even subtypes, or limit the spread of the viruses between pigs and humans, especially for pandemic strains, are therefore needed. Consensus HAs of different strains within one subtype were previously studied for eliciting cross-cluster protection [17], but HA induces predominately a subtype-specific humoral immune response. In contrast, the conserved viral antigens, such as M2e and internal NP, can generate heterosubtypic immunity protective against diverse virus strains and subtypes [18-21]. Unlike HA, the NP-induced cell-mediated immune responses do not prevent infection, but reduce the severity of illness and accelerate the virus clearance. It is reasonable to propose that the combined utility of the consensus HA and conserved viral proteins will confer complete protection against homosubtypic and, at least partial, heterosubtypic challenge. In the present study, two types of swine influenza DNA vaccines were constructed: (1) fusing matrix 2 ectodomain (M2e) to consensus HA of H3 subtype SIV (MHa, means M2e + HA) and, (2) fusing M2e and a conserved CTL epitope, NP147-155, to consensus H3 HA (MNHa, means M2e + NP147-155 + HA). Then their protective efficacy and broadness against divergent H3N2 and H1N1 SIV challenges were tested in mice.

## Results

### The DNA vaccines elicited M2e- and HA-specific antibodies

**Figure 1:**
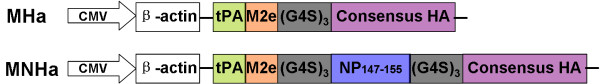
**Schematic diagram of the construct design for two DNA vaccines.** tPA: signal peptide of tissue plasminogen activator; (G4S)_3_: Gly-Gly-Gly-Gly-Ser-Gly-Gly-Gly-Gly-Ser-Gly-Gly-Gly-Gly-Ser linker.

**Figure 2:**
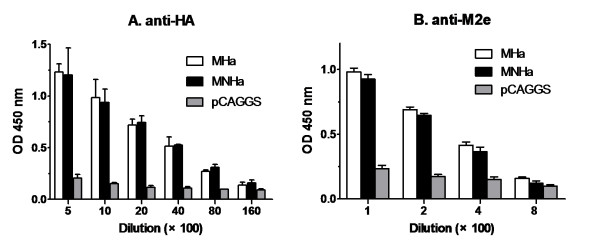
**HA- and M2e-specific antibody response to MHa and MNHa vaccinations.** Mice were immunized twice, three weeks apart, with each construct by using gene gun. Blood were collected 21 days after 2nd shot. ELISA plates were coated with inactivated SwHLJ1 and synthetic M2e peptides, respectively. OD_450_ at each dilution were determined by indirect ELISA.

To increase the expression level, the M2e, CTL epitope and consensus H3 HA gene was optimized for codon usage, RNA structure and GC content. Then the codon-optimized consensus HA, linked with M2e, was cloned into eukaryotic expression vector with or without NP147-155 to generated two DNA vaccines (Figure 1). After nucleotides sequencing, the DNA vaccine plasmids were transfected into human embryonic kidney (HEK) 293 T cells,the expression of each chimeric protein was confirmed by indirect immunofluorescence assay (IFA). IFA showed that both M2e and HA genes expressed *in vitro* (data not shown). Then the two DNA vaccines, as well as empty vector, were coated with gold particles and delivered into the skin with a gene gun. Humoral immunity was analyzed by detecting the presence of antigen-specific antibodies. As can be seen in Figure 2, each of the constructs evoked a substantial HA-specific IgG response after the booster injection, suggesting that the two vaccines were adequately delivered and expressed in mice. No significant difference in serum IgG antibody levels were observed between MHa and MNHa group (*p* > 0.05), indicating that the addition of a 9-mer length CTL epitope did not influence the vaccine’s antibody-inducing ability significantly. But the antibody titer of M2e was lower than that of HA (*p* < 0.001). This is not surprising, because M2e contains only 24 amino acids and it was reported that M2e is less immunogenic than HA [22].

HI assays were then performed on both homosubtypic and heterosubtypic virus to assess the ability of inducing relevant and cross-reactive functional antibodies. As detailed in Table 1, both of the vaccine immunization groups developed HI antibodies against homologous viruses, but induced very low levels of HI antibodies against heterologous viruses.

**Table 1:** Serum HI titers for homologous (H3) and heterologous (H1) strains

	**HI titers (GMT)**			
	**H3 subtype**		**H1 subtype**	
	SwHLJ1	r164	rPan09	G11
MHa	253	320	13	16
MNHa	202	253	10	13
pCAGGS	<10	<10	<10	<10

Sera from each immunized group was pooled, results represent geometric mean titers of 3 independent experiments. Start dilution of sera was 1:10.

### NP147-155 enhanced CTL responses significantly

**Figure 3:**
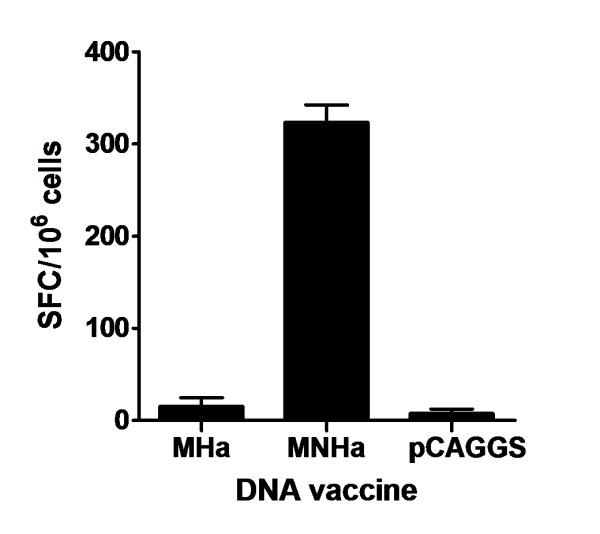
**Number of IFN-γ secreting cells measured by ELISPOT.** Spleen cells were harvest from 3 mice 2 weeks after the last immunization, then pooled and restimulated by peptide of NP147-155. Results were expressed as spot-forming cells (SFC) per million splenocytes. Data are representative of 3 independent experiments.

The ability of the CLT epitope-containing vaccine, MNHa, to induce cellular immune response was determined by IFN-γ ELISPOT assay. A synthesized peptide NP147-155 was used for restimulating splenocytes isolated from 3 mice of each group. Results showed that, after restimulation, the number of activated IFN-γ secreting cells from mice immunized with MNHa was significantly higher than that from mice immunized with MHa and empty vector (*p* < 0.001, Figure 3).

### Both MHa and MNHa vaccination conferred complete protection against homologous challenge, but MNHa showed more significant cross-protection against heterologous challenge than MHa did

**Figure 4:**
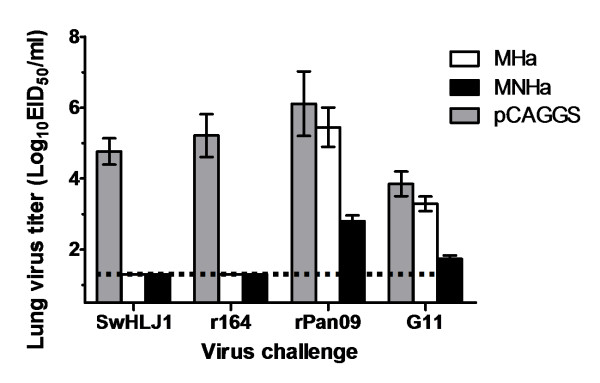
**Protection of mice from homologous and heterologous challenges.** Mice lung viral titers at day 3 after challenges were determined in eggs from an initial dilution of 1:10 in phosphate-buffered saline and expressed as EID_50_/ml. The limit of virus detection was 1.5log.

**Figure 5:**
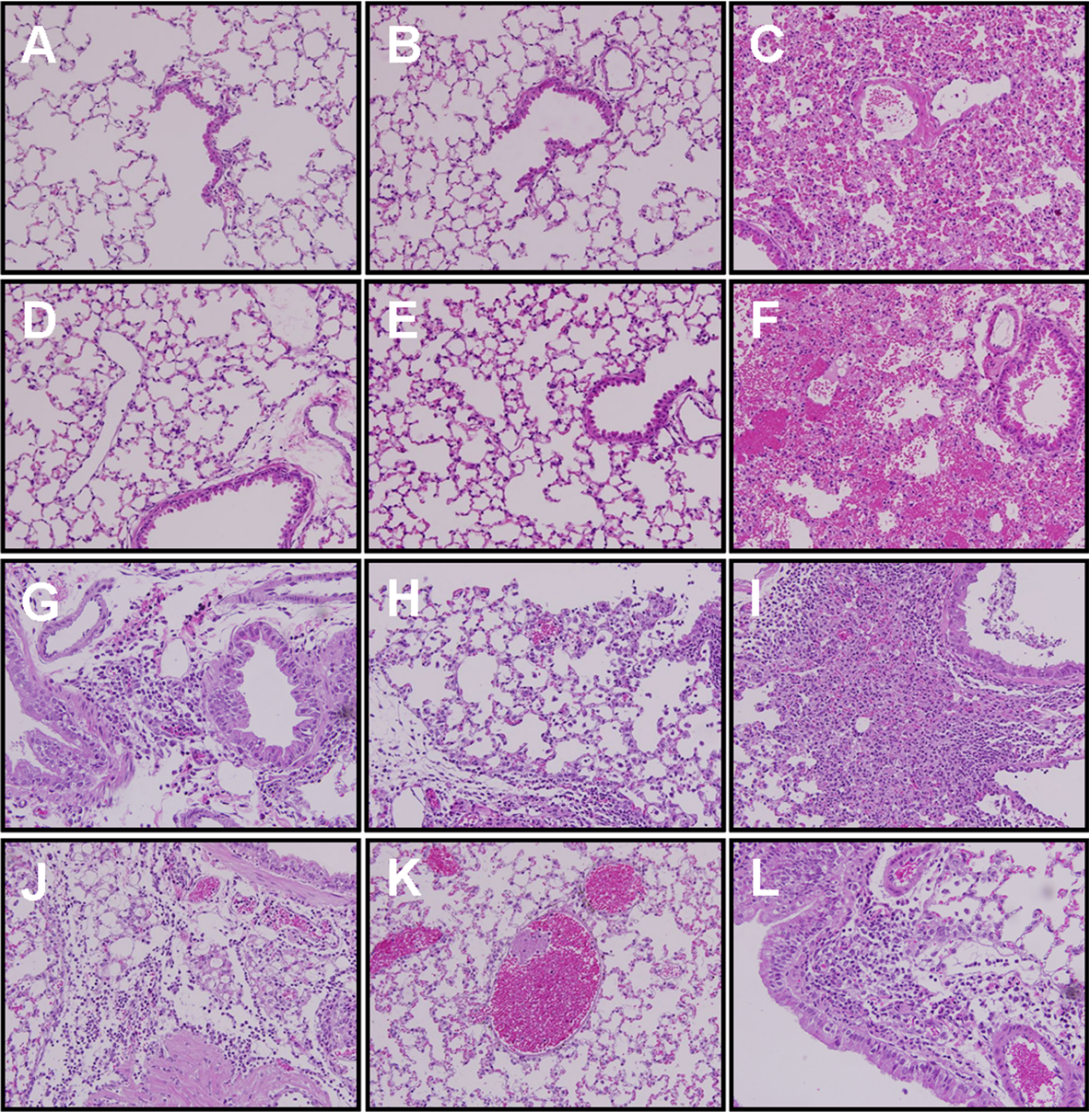
Histopathology analysis of lungs from the mice inoculated with SwHLJ1 (A, B, C), r164 (D, E, F), rPan09 (G, H, I) and G11 (J, K, L) at day 3 post inoculation, after immunized with MHa (A, D, C, J), MNHa (B, E, H, K) and pCAGGS vector (C, F, I, L).

Our previous study indicated that the wild type of SwGD164 strain (H1N1) replicates poorly in mice (unpublished data). To make the virus be suitable for vaccine efficacy assessment *in vivo*, we constructed a recombinant virus (r164) by using reverse genetics. r164 contains HA and NA from SwGD164, and the internal proteins from PR8. BALB/c mice were infected intranasally with two homologous strains belonging to different clusters within H3 subtype 21 days after the last immunization [9]. Mice lungs were taken 3 days post infection for virus titration and histopathologic changes observation. Results demonstrated that there was no detectable virus load in all the vaccine-immunized mice, while empty vector control group showed high lung viral titers (Figure 4). In addition, histopathologic observation showed no obvious histopathologic changes in vaccinated mice. In contrast, the empty vector control group exhibited histopathological damages including the dropout of mucous epithelium cells, interstitial edema, hyperemia, hemorrhage, and inflammatory cell infiltration (Figure 5).

Since M2e and NP147-155 are conserved in different subtypes of influenza viruses, we hypothesized that the DNA vaccine can confer partial protection against heterosubtypic challenge. To test this, the vaccines’ protective ability was studied in mice challenged with H1N1 SIVs, which are also circulating in swine population worldwide. The immunized mice were challenged with heterologous G11 and rPan09, respectively. Lung viral titers determination and pathological analysis were performed as described above. Results indicated that all the mice that had been vaccinated with MHa had a detectable virus level, although showed a reduction in mean viral titers in both challenge groups compared with vector control, the reduction did not reach significance (*p* = 0.06 for rPan09 group and *p* = 0.67 for G11 group, Figure 4). Histopathological analysis exhibited corresponding results, i.e., mice of MHa group had lung pathology as almost severe as vector inoculated group. In MNHa group, although the mice also exhibited detectable virus shedding, the mean viral titers had been significantly reduced in both rPan09- and G11-challenged groups compared to vector-immunized control (*p* < 0.001), showing average viral titer reduction of 3.4 and 2.1 logs, respectively (Figure 4). As expected, pathology damage of mice lung of MNHa group was less pronounced than that of MHa and vector control group, moderate histopathological lesions, mainly vascular congestion and various degrees of hemorrhage were observed (Figure 5). Collectively, these findings indicate consensus HA in combination with M2e elicits cross-cluster immunity, and CTL response contributes to the control and clearance of infection and reduces pathogenesis.

## Discussion

Although the viruses are highly and continuously variable because of antigenic drift and shift, influenza is a vaccine-preventable disease. The conventional inactivated human influenza vaccines are updated annually with the World Health Organization-recommended H1N1, H3N2, and influenza B strains in order to antigenically match the viruses predicted to be the most likely to cause the next epidemic. Unlike human influenza vaccines, strains used for swine influenza vaccines preparation are not regularly replaced, making the vaccines provide limited protection against antigenically diverse SIVs. A universal vaccine designed based on the conserved viral proteins that confers broad protection is an attractive solution to counter the features of highly variable of not only human but also swine influenza viruses. Many studies developed universal vaccines against human influenza, whereas broadly responsive swine influenza vaccines, to our knowledge, have not been reported.

The virus envelope glycoprotein HA is the most abundant surface protein, antibodies against HA can provide protection by blocking virus attachment and entry. Studies from several groups have demonstrated the antiviral efficacy of consensus HA as an “intra-subtype” universal vaccine. The study of Chen et al. [17] showed that the consensus H5 HA-based DNA vaccine elicited only moderate neutralization activities toward the H5N1 clade 2.1 and clade 2.3 viruses, and provided not complete, albeit significant, protection against clade 2.1 virus, suggesting that consensus HA alone may be not enough to induce complete protection against all strains within one subtype, much less heterosubtypic strains. However, ideally, universal vaccines should be capable of inducing protection against both homosubtypic and heterosubtypic challenges. To this end, we linked M2e and CTL-inducing epitope NP147-155 to consensus H3 HA to construct a universal swine influenza vaccine. The reasons we chose M2e and NP147-155 are: (1) both of them are highly conserved among different SIV strains, NP147-155 are even conserved among human, avian and swine influenza strains (Table 2), they were added in view of their ability to broaden the protective scope of the vaccine. (2) NP147-155 induces a vigorous CTL response [23]. Studies indicated that specific CTLs provide some level of cross-protection against antigenically distinct viruses of not only the same subtype but also different subtypes [24,25].

**Table 2:** Amino acids homology of HA, M2e and NP147-155 between the vaccine and challenge strains

**Challenge strain**		**% homology of amino acids**		
		**HA**	**M2e**	**NP147-155**
H1 subtype	rPan09	44.3	79.2	100
	G11	44.0	79.2	100
H3 subtype	SwHLJ1	93.3	91.7	100
	r164	95.1	79.2	100

Previously we have shown that SwHLJ1 and SwGD164 had evolved into 2 independent lineages based on the phylogenetic analysis of the HA gene. To see whether the two strains were antigenically distinct, cross-HI assay was performed by using r164-specific anti-serum. It was found that the titer of cross-reacting HI antibodies (SwHLJ1-reacting) was significantly lower than that of r164-reacting HI antibodies (*p* < 0.001, data not shown), thus suggesting that they belong to different antigenic clusters. Results of present study demontrated that both MHa and MNHa vaccination provided complete protection against these two viruses challenges, cross-cluster protections were therefore suggested. While MNHa, but not MHa, induces immune responses partially protect against heterologous influenza infection, suggesting the echanced T-cell mediated response is not required for homologous protection. Results also further confirm that CTL responses play important roles for heterologous protection, meaning that strengthening CTL responses are promising ways for universal influenza vaccines development.

It was established that the 2009 pandemic H1N1virus is pathogenic and is readily transmitted in pigs [26-29]. Although the new H1N1 virus is now considered to be post-pandemic, the possibility that it recombines with other influenza viruses in pigs then yielding a novel potential epidemic or pandemic strain still exists[30]. Here in this study, the mice were partially protected from the reassortant 2009 pandemic virus challenge after the immunization of MNHa. The vaccine immunization inhibited the pulmonary viral replication significantly in mice, thus could accelerate the virus clearance, and reduce the potential of transmission and the risk of recombination. Although the heterologous protection elicited by the DNA vaccine was not complete, this methodology has a large potential for improvements. The present study lays the foundation for universal swine influenza vaccine development, and call for further investigations in which the heterologous immune response should be further enhanced, such as the addition of molecular adjuvants [31,32] and/or more copies of conserved viral protein encoding genes [33], and the usage of DNA-prime protein/virus-boost immunization schedule [34,35]. A previous study conducted by Heinen et al. constructed a DNA vaccine containing M2e and full length of NP genes, but HA encoding gene was not included. Their data showed that the M2e-NP DNA vaccination produced enhancement of disease after challenge [36]. Although the mechanism underlying which is unclear, we think the HA may play roles in this discrepancy between the results of Heinen et al.’s and ours. Another possible reason should be taken into consideration is that gene gun immunization induces more strong immune response that the conventional needle injection method, which was used in Heinen et al.’s study [37]. It is necessary to study whether the DNA vaccines we developed here will confer protection in pig models. Also, it would be interesting to study how, if does, the HA protein influence the vaccines’ efficacy.

## Conclusions

The combined utility of the consensus HA and the conserved M2e and CTL epitope can confer complete and partial protection against homologous and heterologous challenges, respectively, in mouse models. This may provide a promising strategy for universal swine influenza vaccine development.

## Materials and methods

### Viruses, cell and mice

Swine influenza viruses of A/Swine/Heilongjing/1/05 (H3N2) [SwHLJ1], A/Swine/Guangdong/164/06 (H3N2) [SwGD164] and A/Swine/Guangdong/1/01(H1N1) [G11] were isolated previously by us [6]. Briefly, clinical samples from diseased pigs were homogenized in phosphate-buffered saline (PBS) containing antibiotics, then inoculated into allantoic cavities of specific pathogen free (SPF) embryonated chicken eggs, the eggs were incubated for at least 48 h at 35°C. Virus isolate was passaged and identified by hemagglutination inhibition (HI) test and neuraminidase inhibition (NI) test using a panel of reference sera. Reassortant virus with surface glycoproteins of 2009 pandemic A/Califorina/04/2009 and internal proteins of PR8 virus (abbreviated as rPan09) was kindly provided by Drs. Wen-Bao Qi and Ming Liao of College of Veterinary Medicine, South China Agricultural University. r164, containing SwGD164 surface glycoproteins and PR8 internal proteins, was prepared by using reverse genetics as described [38]. These viruses were propagated in allantoic cavities of 9 to 10-day old SPF embryonated chicken eggs and stored at −80°C until use. Fifty-percent embryo infectious dose (EID_50_) titers were determined by serial titration in embryos and calculated by the method of Reed and Muench [39]. HEK 293 T cells were maintained in Dulbecco's modified Eagle's medium (DMEM, Sigma) supplemented with 10% fetal bovine serum in humidified 5% CO_2_ atmosphere at 37°C. SPF female BALB/c mice (4–6 weeks of age) were purchased from Shanghai SLAC Laboratory Animal Co., Ltd., and maintained with free access to sterile food and water. All animal studies were conducted in accordance with the ethical guidelines and were approved by the Ethical Committee of Shanghai Veterinary Research Institute, Chinese Academy of Agricultural Sciences.

### Plasmid construction

A total of 162 HA and 105 M2 sequences with full-length of H3N2 subtype SIV were, respectively, downloaded from GenBank database and aligned by MegAlign program supplemented in DNASTAR package (DNASTAR Madison, WI). The consensus sequences were created based on the most common amino acid in each position of the alignment. After the consensus M2 sequence was generated, the first 24 amino acids were selected as M2e motif [40]. Then codons of the consensus HA, as well as M2e and an immunodominant cytotoxic T lymphocyte (CTL) epitope of the NP protein, NP147-155 (TYQRTRALV) [41], were optimized for mammalian expression and synthesized by GenScript Corporation (Nanjing, China). The (G4S)3-linked chimeric ORF encoding M2e, CTL epitope and consensus HA was PCR-amplified by introducing 5’ *Sma* I and 3’ *Nhe* I restriction sites for ligation into the pCAGGS vector, under the control of the cytomegalovirus (CMV) enhancer and chicken β-actin promoter (designated as MNHa). Similarly, MHa was designed and constructed, except that the CTL epitope was omitted from the N-terminal end of the consensus HA (Figure 1). After the recombinant plasmids being identified by nucleotide sequencing, they were propagated in *E. coli* bacteria and purified using Mega purification kit (Qiagen, Valencia, CA) for *in vitro* transfection as well as *in vivo* animal immunization. The final DNA preparations were resuspended in nuclease-free water and stored at −20°C until further use.

### *In vitro* expression of recombinant protein

HEK 293 T cells were seeded in 6-well plates and transfected at 80-90% confluence with 4 μg of MHa, MNHa or empty vector using Lipofectamine 2000 transfection reagent (Invitrogen), as recommended by the manufacturer. After 48 h, the transfected cells were scraped from the culturing plate, washed with PBS, then spotted onto a glass slide, air dried, and fixed with pre-chilled acetone. Upon removal of the residual solvents from the slides, the cells were incubated with anti-M2e and -HA polyclonal antibodies for 1 h at 37°C. A secondary Alexa Fluor 568-conjugated goat anti-mouse IgG antibody was then used to detect the primary antibody. Fluorescence images were scanned using an inverted microscope after the samples were mounted by glycerol.

### Gene gun delivery of DNA and virus challenge

Gene gun immunization was performed as previously described [42,43]. Mice received two nonoverlapping abdominal deliveries of antigen encoding plasmid- or empty vector-coated gold beads (1 μm) at the shaved skin with a 3-wk interval. With each shot, 2 μg of DNA immobilized onto 0.5 mg gold particles was delivered at a helium discharge pressure of 450–500 psi with a Helios gene gun (Bio-Rad). Each test group contained 43 mice with experiments organized as follows: (1) 3 mice of each group were used to complete IFN-γ ELISPOT assays on day 21 post the 2nd immunization; (2) the left 40 mice of each group were divided as 4 subgroups, each comprised of 10 mice, then intranasally challenged with 10^6^ EID_50_ of two H3N2 (SwHLJ1 and r164) and two H1N1 (G11 and rPan09) strains, respectively, at 21 days after the final immunization.

### Serologic testing

Serum samples were collected from orbital bleeds on day 14 post last immunization. M2e and HA antibody titers were measured using indirect ELISA. ELISA plates were coated overnight with synthetic M2e peptides or inactivated SwHLJ1 in 0.1 M carbonate buffer (pH 9.6) at 4°C and blocked with 5% non-fat milk for 2 h at 37°C. A series of two-fold dilution of sera (starting dilution 1:500 for HA and 1:100 for M2e, 100 μl/well) were incubated at 37°C for 1 h, followed by three washes with PBST. Then HRP-conjugated goat anti-mouse IgG (Zhongshan Biotechnology Co., Ltd Beijing, China) and 3,3'-5,5'-tetramethyl benzidine (TMB) was used for M2- and HA-specific antibody detection and color development, respectively. The resulting optical density (OD) at 450 nm was determined with a plate reader after the reaction was stopped with 2 M H_2_SO_4_.

HI assays were performed using 0.5% chicken red blood cells (RBCs) with 4 HA units of homologous (SwHLJ1 and r164) and heterologous (G11 and rPan09) virus and receptor-destroying enzyme (RDE, sigma) treated serum, as previously described [44]. The reciprocal of highest dilution of serum that gave complete inhibition of hemagglutination was considered the HI titer. Each assay was performed in triplicate. A titer of less than 10 (starting serum dilution) was assigned for serum samples that did not inhibit hemagglutination.

### IFN-γ ELISPOT

The ELISPOT assays were performed using mouse IFN-γ ELISPOT kits following methods recommended by the manufacturer (Dakewe Biotech, PR China). Briefly, single-cell suspensions of freshly isolated spleen lymphocytes were seeded into the plates (10^6^/well) pre-coated with anti-IFN-γ monoclonal antibody. Cells were stimulated with synthesized NP147-155 peptide at a final concentration of 5 μg/ml in a 37°C humidified incubator with 5% CO_2_. Phytohemagglutinin (PHA, 5 μg/ml) and medium alone were used as positive and negative controls, respectively. After a 36 h culture, plates were washed and incubated for 1 h with biotinylated anti-mouse IFN-γ antibody. Streptavidin-horseradish peroxidase was then added, IFN-γ spots were developed with 3-amino-9-ethylcarbazole (AEC) and counted using an automated ELISPOT reader. Results were expressed as spot-forming cells (SFC) per million cells.

### Lung viral titer measurement and histopathological examination

On day 3 post-infection, mice from each group were sacrificed to collect lungs for virus titration and pathologic examination to determine the protective ability of DNA vaccines. For virus titration, the whole lungs were homogenized in 1 mL of sterile phosphate-buffered saline (PBS) containing 0.1 mg/ml of streptomycin and 100 IU/ml of penicillin. The homogenates were diluted 10-fold serially after being centrifuged, then inoculated into embryonated chicken eggs. Infection within individual eggs was confirmed using a standard hemagglutination assay, the viral titers were determined by the method of Reed and Muench.

Lung tissue histopathologic sections were made as described elsewhere [45]. Briefly, the removed lungs were fixed in 10% neutral buffered formalin, dehydrated and embedded in paraffin. Five micrometer sections were cut and stained with hematoxylin and eosin (H&E), and reviewed for histopathologic changes.

### Statistical analysis

Data of experimental and control groups were presented as the averages ± standard error (SE) and evaluated by ANOVA method, where statistically significant results were defined as having a *p* value of less than 0.05.

## Competing interests

The authors declare that they have no competing interests.

## Authors’ contributions

BW and GZT conceived the study and wrote the paper. BW, HY, FRY, MH and JHM performed the experiments. BW analyzed the data. All authors have read and approved the final manuscript.

## References

[B1] Van Reeth K Nauwynck H Pensaert M Dual infections of feeder pigs with porcine reproductive and respiratory syndrome virus followed by porcine respiratory coronavirus or swine influenza virus: a clinical and virological study Vet Microbiol 1996 48 325 335 10.1016/0378-1135(95)00145-X 9054128 PMC7117459

[B2] Ito T Couceiro JN Kelm S Baum LG Krauss S Castrucci MR Donatelli I Kida H Paulson JC Webster RG Kawaoka Y Molecular basis for the generation in pigs of influenza A viruses with pandemic potential J Virol 1998 72 7367 7373 9696833 10.1128/jvi.72.9.7367-7373.1998 PMC109961

[B3] Neumann G Noda T Kawaoka Y Emergence and pandemic potential of swine-origin H1N1 influenza virus Nature 2009 459 931 939 10.1038/nature08157 19525932 PMC2873852

[B4] Dawood FS Jain S Finelli L Shaw MW Lindstrom S Garten RJ Gubareva LV Xu X Bridges CB Uyeki TM Emergence of a novel swine-origin influenza A (H1N1) virus in humans N Engl J Med 2009 360 2605 2615 19423869 10.1056/NEJMoa0903810

[B5] Macklin MD McCabe D McGregor MW Neumann V Meyer T Callan R Hinshaw VS Swain WF Immunization of pigs with a particle-mediated DNA vaccine to influenza A virus protects against challenge with homologous virus J Virol 1998 72 1491 1496 9445052 10.1128/jvi.72.2.1491-1496.1998 PMC124630

[B6] Yu H Zhang GH Hua RH Zhang Q Liu TQ Liao M Tong GZ Isolation and genetic analysis of human origin H1N1 and H3N2 influenza viruses from pigs in China Biochem Biophys Res Commun 2007 356 91 96 10.1016/j.bbrc.2007.02.096 17346674

[B7] Guan Y Shortridge KF Krauss S Li PH Kawaoka Y Webster RG Emergence of avian H1N1 influenza viruses in pigs in China J Virol 1996 70 8041 8046 8892928 10.1128/jvi.70.11.8041-8046.1996 PMC190877

[B8] Peiris JS Guan Y Markwell D Ghose P Webster RG Shortridge KF Cocirculation of avian H9N2 and contemporary "human" H3N2 influenza A viruses in pigs in southeastern China: potential for genetic reassortment? J Virol 2001 75 9679 9686 10.1128/JVI.75.20.9679-9686.2001 11559800 PMC114539

[B9] Yu H Hua RH Zhang Q Liu TQ Liu HL Li GX Tong GZ Genetic evolution of swine influenza A (H3N2) viruses in China from 1970 to 2006 J Clin Microbiol 2008 46 1067 1075 10.1128/JCM.01257-07 18199784 PMC2268354

[B10] Richt JA Lager KM Janke BH Woods RD Webster RG Webby RJ Pathogenic and antigenic properties of phylogenetically distinct reassortant H3N2 swine influenza viruses cocirculating in the United States J Clin Microbiol 2003 41 3198 3205 10.1128/JCM.41.7.3198-3205.2003 12843064 PMC165376

[B11] Webby RJ Rossow K Erickson G Sims Y Webster R Multiple lineages of antigenically and genetically diverse influenza A virus co-circulate in the United States swine population Virus Res 2004 103 67 73 10.1016/j.virusres.2004.02.015 15163491

[B12] Vincent AL Lager KM Ma W Lekcharoensuk P Gramer MR Loiacono C Richt JA Evaluation of hemagglutinin subtype 1 swine influenza viruses from the United States Vet Microbiol 2006 118 212 222 10.1016/j.vetmic.2006.07.017 16962262

[B13] Bikour MH Cornaglia E Elazhary Y Evaluation of a protective immunity induced by an inactivated influenza H3N2 vaccine after an intratracheal challenge of pigs Can J Vet Res 1996 60 312 314 8904668 PMC1263854

[B14] de Jong JC Heinen PP Loeffen WL van Nieuwstadt AP Claas EC Bestebroer TM Bijlsma K Verweij C Osterhaus AD Rimmelzwaan GF Antigenic and molecular heterogeneity in recent swine influenza A(H1N1) virus isolates with possible implications for vaccination policy Vaccine 2001 19 4452 4464 10.1016/S0264-410X(01)00190-6 11483271

[B15] Van Reeth K Gregory V Hay A Pensaert M Protection against a European H1N2 swine influenza virus in pigs previously infected with H1N1 and/or H3N2 subtypes Vaccine 2003 21 1375 1381 10.1016/S0264-410X(02)00688-6 12615433

[B16] Vincent AL Ciacci-Zanella JR Lorusso A Gauger PC Zanella EL Kehrli ME Janke BH Lager KM Efficacy of inactivated swine influenza virus vaccines against the 2009 A/H1N1 influenza virus in pigs Vaccine 2010 28 2782 2787 10.1016/j.vaccine.2010.01.049 20132919

[B17] Chen MW Cheng TJ Huang Y Jan JT Ma SH Yu AL Wong CH Ho DD A consensus-hemagglutinin-based DNA vaccine that protects mice against divergent H5N1 influenza viruses Proc Natl Acad Sci U S A 2008 105 13538 13543 10.1073/pnas.0806901105 18765801 PMC2533225

[B18] Neirynck S Deroo T Saelens X Vanlandschoot P Jou WM Fiers W A universal influenza A vaccine based on the extracellular domain of the M2 protein Nat Med 1999 5 1157 1163 10.1038/13484 10502819

[B19] Tompkins SM Zhao ZS Lo CY Misplon JA Liu T Ye Z Hogan RJ Wu Z Benton KA Tumpey TM Epstein SL Matrix protein 2 vaccination and protection against influenza viruses, including subtype H5N1 Emerg Infect Dis 2007 13 426 435 10.3201/eid1303.061125 17552096 PMC2725899

[B20] Rao SS Kong WP Wei CJ Van Hoeven N Gorres JP Nason M Andersen H Tumpey TM Nabel GJ Comparative efficacy of hemagglutinin, nucleoprotein, and matrix 2 protein gene-based vaccination against H5N1 influenza in mouse and ferret PLoS One 2010 5 e9812 10.1371/journal.pone.0009812 20352112 PMC2843722

[B21] Jimenez GS Planchon R Wei Q Rusalov D Geall A Enas J Lalor P Leamy V Vahle R Luke CJ Vaxfectin-formulated influenza DNA vaccines encoding NP and M2 viral proteins protect mice against lethal viral challenge Hum Vaccin 2007 3 157 164 10.4161/hv.3.5.4175 17637571

[B22] Lamb RA Zebedee SL Richardson CD Influenza virus M2 protein is an integral membrane protein expressed on the infected-cell surface Cell 1985 40 627 633 10.1016/0092-8674(85)90211-9 3882238

[B23] Ulmer JB Donnelly JJ Parker SE Rhodes GH Felgner PL Dwarki VJ Gromkowski SH Deck RR DeWitt CM Friedman A Heterologous protection against influenza by injection of DNA encoding a viral protein Science 1993 259 1745 1749 10.1126/science.8456302 8456302

[B24] Adar Y Singer Y Levi R Tzehoval E Perk S Banet-Noach C Nagar S Arnon R Ben-Yedidia T A universal epitope-based influenza vaccine and its efficacy against H5N1 Vaccine 2009 27 2099 2107 10.1016/j.vaccine.2009.02.011 19356612

[B25] Sui Z Chen Q Wu R Zhang H Zheng M Wang H Chen Z Cross-protection against influenza virus infection by intranasal administration of M2-based vaccine with chitosan as an adjuvant Arch Virol 2010 155 535 544 10.1007/s00705-010-0621-4 20195654

[B26] Itoh Y Shinya K Kiso M Watanabe T Sakoda Y Hatta M Muramoto Y Tamura D Sakai-Tagawa Y Noda T In vitro and in vivo characterization of new swine-origin H1N1 influenza viruses Nature 2009 460 1021 1025 19672242 10.1038/nature08260 PMC2748827

[B27] Lange E Kalthoff D Blohm U Teifke JP Breithaupt A Maresch C Starick E Fereidouni S Hoffmann B Mettenleiter TC Pathogenesis and transmission of the novel swine-origin influenza virus A/H1N1 after experimental infection of pigs J Gen Virol 2009 90 2119 2123 10.1099/vir.0.014480-0 19592456

[B28] Weingartl HM Albrecht RA Lager KM Babiuk S Marszal P Neufeld J Embury-Hyatt C Lekcharoensuk P Tumpey TM Garcia-Sastre A Richt JA Experimental infection of pigs with the human 1918 pandemic influenza virus J Virol 2009 83 4287 4296 10.1128/JVI.02399-08 19224986 PMC2668479

[B29] Vincent AL Lager KM Faaberg KS Harland M Zanella EL Ciacci-Zanella JR Kehrli ME Janke BH Klimov A Experimental inoculation of pigs with pandemic H1N1 2009 virus and HI cross-reactivity with contemporary swine influenza virus antisera Influenza Other Respi Viruses 2010 4 53 60 10.1111/j.1750-2659.2009.00121.x 20167045 PMC5779285

[B30] Zhu H Zhou B Fan X Lam TT Wang J Chen A Chen X Chen H Webster RG Webby R Novel reassortment of Eurasian Avian-like and pandemic/2009 influenza viruses in swine: infectious potential to humans J Virol 2011 10.1128/JVI.05352-11 PMC3187487 21849442

[B31] Larsen DL Dybdahl-Sissoko N McGregor MW Drape R Neumann V Swain WF Lunn DP Olsen CW Coadministration of DNA encoding interleukin-6 and hemagglutinin confers protection from influenza virus challenge in mice J Virol 1998 72 1704 1708 9445082 10.1128/jvi.72.2.1704-1708.1998 PMC124660

[B32] Luke JM Simon GG Soderholm J Errett JS August JT Gale M Hodgson CP Williams JA Coexpressed RIG-I Agonist Enhances Humoral Immune Response to Influenza Virus DNA Vaccine J Virol 2011 85 1370 1383 10.1128/JVI.01250-10 21106745 PMC3020507

[B33] Park KS Seo YB Lee JY Im SJ Seo SH Song MS Choi YK Sung YC Complete protection against a H5N2 avian influenza virus by a DNA vaccine expressing a fusion protein of H1N1 HA and M2e Vaccine 2011 29 5481 5487 10.1016/j.vaccine.2011.05.062 21664216

[B34] Larsen DL Karasin A Olsen CW Immunization of pigs against influenza virus infection by DNA vaccine priming followed by killed-virus vaccine boosting Vaccine 2001 19 2842 2853 10.1016/S0264-410X(01)00014-7 11282195

[B35] Le Gall-Recule G Cherbonnel M Pelotte N Blanchard P Morin Y Jestin V Importance of a prime-boost DNA/protein vaccination to protect chickens against low-pathogenic H7 avian influenza infection Avian Dis 2007 51 490 494 10.1637/7592-040206R.1 17494616

[B36] Heinen PP Rijsewijk FA de Boer-Luijtze EA Bianchi AT Vaccination of pigs with a DNA construct expressing an influenza virus M2-nucleoprotein fusion protein exacerbates disease after challenge with influenza A virus J Gen Virol 2002 83 1851 1859 12124449 10.1099/0022-1317-83-8-1851

[B37] Wang S Zhang C Zhang L Li J Huang Z Lu S The relative immunogenicity of DNA vaccines delivered by the intramuscular needle injection, electroporation and gene gun methods Vaccine 2008 26 2100 2110 10.1016/j.vaccine.2008.02.033 18378365 PMC2790191

[B38] Hoffmann E Neumann G Kawaoka Y Hobom G Webster RG A DNA transfection system for generation of influenza A virus from eight plasmids Proc Natl Acad Sci U S A 2000 97 6108 6113 10.1073/pnas.100133697 10801978 PMC18566

[B39] Reed LJ Muench HA A simple method of estimating fifty per cent endpoints Am J Hyg 1938 37 493 497

[B40] Liu W Zou P Ding J Lu Y Chen YH Sequence comparison between the extracellular domain of M2 protein human and avian influenza A virus provides new information for bivalent influenza vaccine design Microbes Infect 2005 7 171 177 10.1016/j.micinf.2004.10.006 15777646

[B41] Bodmer HC Pemberton RM Rothbard JB Askonas BA Enhanced recognition of a modified peptide antigen by cytotoxic T cells specific for influenza nucleoprotein Cell 1988 52 253 258 10.1016/0092-8674(88)90514-4 2449284

[B42] Huber VC McKeon RM Brackin MN Miller LA Keating R Brown SA Makarova N Perez DR Macdonald GH McCullers JA Distinct contributions of vaccine-induced immunoglobulin G1 (IgG1) and IgG2a antibodies to protective immunity against influenza Clin Vaccine Immunol 2006 13 981 990 10.1128/CVI.00156-06 16960108 PMC1563571

[B43] Conry RM Widera G LoBuglio AF Fuller JT Moore SE Barlow DL Turner J Yang NS Curiel DT Selected strategies to augment polynucleotide immunization Gene Ther 1996 3 67 74 8929913

[B44] Webster RG Kawaoka Y Taylor J Weinberg R Paoletti E Efficacy of nucleoprotein and haemagglutinin antigens expressed in fowlpox virus as vaccine for influenza in chickens Vaccine 1991 9 303 308 10.1016/0264-410X(91)90055-B 1651609

[B45] Belser JA Wadford DA Pappas C Gustin KM Maines TR Pearce MB Zeng H Swayne DE Pantin-Jackwood M Katz JM Tumpey TM Pathogenesis of pandemic influenza A (H1N1) and triple-reassortant swine influenza A (H1) viruses in mice J Virol 2010 84 4194 4203 10.1128/JVI.02742-09 20181710 PMC2863721

